# Identification of genetic loci jointly influencing COVID-19 and coronary heart diseases

**DOI:** 10.1186/s40246-023-00547-8

**Published:** 2023-11-14

**Authors:** Siyue Wang, Hexiang Peng, Feng Chen, Chunfang Liu, Qiwen Zheng, Mengying Wang, Jiating Wang, Huan Yu, Enci Xue, Xi Chen, Xueheng Wang, Meng Fan, Xueying Qin, Yiqun Wu, Jin Li, Ying Ye, Dafang Chen, Yonghua Hu, Tao Wu

**Affiliations:** 1 Department of Epidemiology and Biostatistics, School of Public Health, Peking University, Beijing, 100191 China; 2 Department of Intensive Care Unit, PLA Rocket Force Characteristic Medical Center, Beijing, 100088 China; 3 School of Public Health, Baotou Medical College, Baotou, 014040 China; 4 CAS Key Laboratory of Genomic and Precision Medicine, Beijing Institute of Genomics, Chinese Academy of Sciences, Beijing, 100101 China; 5 China National Center for Bioinformation, Beijing, 100101 China; 6 Department of Local Diseases Control and Prevention, Fujian Provincial Center for Disease Control and Prevention, Fuzhou, 350001 China

## Abstract

**Background:**

Comorbidities of coronavirus disease 2019 (COVID-19)/coronary heart disease (CHD) pose great threats to disease outcomes, yet little is known about their shared pathology. The study aimed to examine whether comorbidities of COVID-19/CHD involved shared genetic pathology, as well as to clarify the shared genetic variants predisposing risks common to COVID-19 severity and CHD risks.

**Methods:**

By leveraging publicly available summary statistics, we assessed the genetically determined causality between COVID-19 and CHD with bidirectional Mendelian randomization. To further quantify the causality contributed by shared genetic variants, we interrogated their genetic correlation with the linkage disequilibrium score regression method. Bayesian colocalization analysis coupled with conditional/conjunctional false discovery rate analysis was applied to decipher the shared causal single nucleotide polymorphisms (SNPs).

**Findings:**

Briefly, we observed that the incident CHD risks post COVID-19 infection were partially determined by shared genetic variants. The shared genetic variants contributed to the causality at a proportion of 0.18 (95% CI 0.18–0.19) to 0.23 (95% CI 0.23–0.24). The SNP (rs10490770) located near *LZTFL1* suggested direct causality (SNPs → COVID-19 → CHD), and SNPs in *ABO* (rs579459, rs495828), *ILRUN*(rs2744961)*,* and *CACFD1*(rs4962153, rs3094379) may simultaneously influence COVID-19 severity and CHD risks.

**Interpretation:**

Five SNPs located near *LZTFL1* (rs10490770)*, ABO* (rs579459, rs495828), *ILRUN* (rs2744961), and *CACFD1 *(rs4962153, rs3094379) may simultaneously influence their risks. The current study suggested that there may be shared mechanisms predisposing to both COVID-19 severity and CHD risks. Genetic predisposition to COVID-19 is a causal risk factor for CHD, supporting that reducing the COVID-19 infection risk or alleviating COVID-19 severity among those with specific genotypes might reduce their subsequent CHD adverse outcomes. Meanwhile, the shared genetic variants identified may be of clinical implications for identifying the target population who are more vulnerable to adverse CHD outcomes post COVID-19 and may also advance treatments of ‘Long COVID-19.’

**Supplementary Information:**

The online version contains supplementary material available at 10.1186/s40246-023-00547-8.

## Introduction

Severe acute respiratory syndrome coronavirus 2 (SARS-CoV-2) is responsible for coronavirus disease 2019 (COVID-19) and the current global pandemic. It has soon gone virus across the world, affecting more than 200 countries/territories [1]. To date, the world has registered more than 24 million individuals contaminated, with more than 5 million deaths [2]. While the disease has mild effects in most individuals, severe COVID-19 is more likely to be observed in the those with comorbidities such as cardiovascular diseases [3]. Additionally, why certain populations are at a higher risk of adverse CHD outcomes post COVID-19 infection is still unclear [4].

Accumulating evidence revealed a bidirectional relationship between coronary heart disease (CHD) and COVID-19 [5–8], yet consensus has not been achieved regarding their causality. Patients of cardiovascular diseases are at higher risk of severe COVID-19 and death [3, 9–11]. Meanwhile, CHD complications are observed in patients recovering from COVID-19. However, little is known as to whether COVID-19 infections causally induce CHD that were not in existence prior to the infection, or vice versa [8, 12]. From an ethical perspective, causal inference with a randomization clinical trial (RCT) is almost infeasible, as it is unethical to leave patients with one disease untreated with the aim of observing the occurrence of another disease. Therefore, it is anticipated to assess the causality between COVID-19 and CHD with an alternative method, for example, bidirectional Mendelian randomization (MR) [13]. Through leverage of randomly allocated genetic variants, bidirectional MR is expected to overcome the issues arising from ethical perspectives, as well as the confounders that hinder causal inference from observational studies [14, 15].

Observational studies indicated that COVID-19 and CHD may share common genetic variants [16, 17]. Suggestively, several genome-wide association studies (GWASs) identified certain SNPs responsible for COVID-19 susceptibility, including *ACE2* and *ABO* [19, 20], which were also found to be associated with CHD risks [18–20]. However, limited studies to date provided with a comprehensive picture of where these shared genetic variants lied in a genome-wide scale. It is thus anticipated to advance the current knowledge of their underlying mechanism by systematically locating these shared genetic variants.

The current study aimed to assess the causality between COVID-19 and CHD, as well as to clarify their shared genetic SNPs.

## Methods

### Study pipeline and data sources

**Fig. 1:**
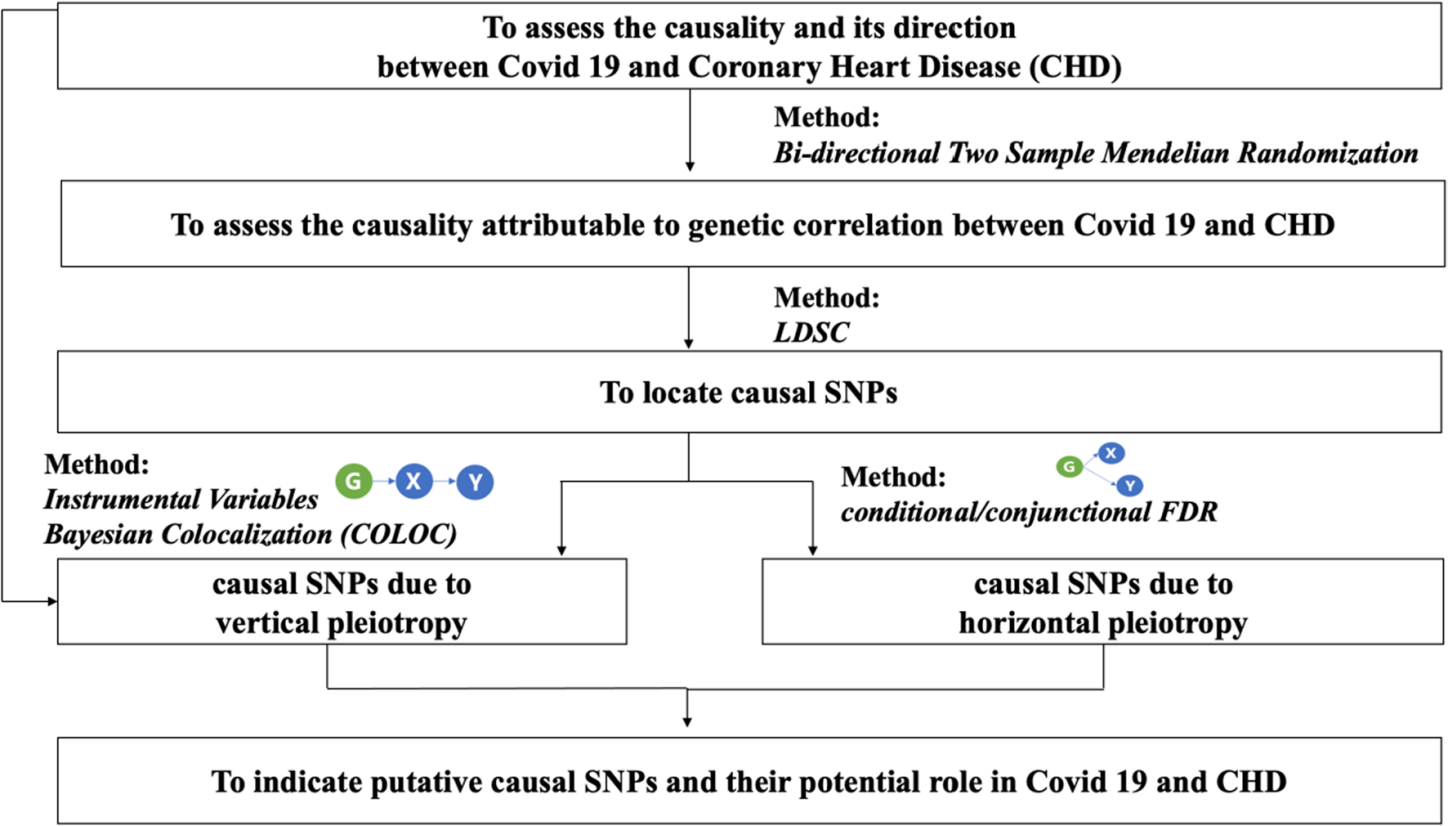
The study pipeline to investigate putative COVID-19-mediated causal pathways to CHD. * X and Y in blue circles are nonspecific designations for either COVID-19 or CHD, depending on previous results from bidirectional Mendelian randomization. G in green circles are putative causal SNPs (either of vertical pleiotropy or horizontal pleiotropy) to be identified

Figure 1 depicts the study pipeline. First, causality and its direction were assessed with a bidirectional MR. Second, to quantify the contribution from the shared genetic variants, we utilized the linkage disequilibrium score regression (LDSC method. Third, we applied the Bayesian colocalization (COLOC) and conditional/conjunctional false discovery rate (cond/conj FDR) to locate shared causal SNPs. For distinguishing SNPs of vertical pleiotropy (SNP → Trait1 → Trait2) with horizontal pleiotropy (SNP simultaneously influence Trait1&2), we searched biological pathway databases to examine whether SNPs were involved in multiple pathways [30].

We leveraged publicly available GWAS summary statistics of COVID-19 and CHD to perform the analyses. A detailed description of the GWAS profiles was presented in Additional file 1: Table S1. Specifically, the GWAS summary statistics of COVID-19 infections were drawn from the COVID-19 Host Genetic Initiative (COVID-19 HG) (https://www.covid19hg.org/results/r5/). The COVID-19 Host Genetics Initiative is an international collaboration aimed at uncovering the genetic determinants of COVID-19 susceptibility and severity. To achieve this goal, researchers collected individual-level clinical and genetic data and conducted individual GWAS. All participating cohorts imputed genotypes to the Haplotype Reference Consortium, 1000 Genomes, or TOPMed reference panels. Each cohort categorized ancestry through self-report or genetic data and performed single-variant association testing while adjusting for covariates such as age, age^2^, sex, age × sex, genetic ancestry principal components, and study-specific factors. Its round 5 release provided summary statistics of COVID-19 genetic susceptibility of differentiated severity among Europeans. COVID-19 genetic susceptibility was investigated in 3 different datasets, namely, COVID-19_A defined as the very severe respiratory confirmed COVID-19 versus the general population (5101 cases/1,383,241 controls), COVID-19_B defined as the hospitalized versus the general population (9986 cases/1,877,672 controls), and COVID-19_C defined as a positive COVID-19 diagnosis versus the general population (38,984 cases/1,644,784 controls). Normally, these 3 datasets assessed the genetic susceptibility to COVID-19 of differentiated severity [21].

The CHD GWAS summary statistic was acquired from the CARDIoGRAMplusC4D consortium (http://www.cardiogramplusc4d.org/), contributed by CARDIoGRAMplusC4D investigators, which currently possesses the largest publicly available CHD GWAS meta-analysis results for Europeans [22]. The CHD dataset (CARDIoGRAM GWAS) comprised 22,233 CHD cases and 64,762 controls from 22 case–control studies [23]. Case status was defined by an inclusive CHD diagnosis (e.g., myocardial infarction, acute coronary syndrome, chronic stable angina, or coronary stenosis > 50%). Each study was analyzed separately under additive logistic regression, and the results were merged by meta-analysis using an inverse-variance weighted fixed-effects model [23]. We applied the LiftOver tool (http://genome.ucsc.edu/cgi-bin/hgLiftOver) to flip CHD GWAS build to GRCh37, so as to align with the COVID-19 coordinate.

## Bidirectional mendelian randomization

We applied bidirectional MR to test whether COVID-19 causally affected CHD risks or vice versa, where either COVID-19-associated SNPs or CHD-associated SNPs (*P* < 5 × 10^–5^) were used as instrumental variables (IVs). We further clumped the IVs at a linkage disequilibrium threshold of 0.2 (*r*^2^ < 0.2) within a distance of 5000 kb. Finally, the SNPs for being palindromic with intermediate allele frequencies were removed when harmonized with the variants.

Of the four MR methods applied in this study, the inverse-variance-weighted (IVW) method was performed in the primary analysis [24]. Additional sensitivity analyses, namely, the MR-Egger, the weighted mode and the simple mode method, were performed to test for robustness of the causality. The MR-Egger method was applied to control potential bias in cases of invalid or weak IVs, which was reported to be capable of controlling the pleiotropic effect of genetic variants that is not mediated via exposure [25]. Similarly, the weighted mode and simple mode were both applied to reduce invalid or weak instrument bias [26, 27]. To account for the potential influence of BMI and T2D on the association between COVID-19 and CHD, we conducted multivariable mendelian randomization analyses using GWAS summary statistics from the IEU open GWAS project (https://gwas.mrcieu.ac.uk/datasets/). GWAS summary statistics for BMI were derived from the UK Biobank (*N* = 461,460, GWAS id: ukb-b-19953), and those for T2D were obtained from another European study with a sample size of *N* = 655,666 (GWAS id: ebi-a-GCST006867).

## Linkage disequilibrium score regression

To quantify the contribution from shared genetic variants of COVID-19 and CHD, we used the LDSC method (https://github.com/bulik/ldsc) [28, 29]. The genetic overlap laid basis for further locating causal SNPs. To examine the genetic overlap across the whole genome, we considered the effects of all SNPs, with uncorrected *P* values [30]. European ancestry information from the 1000 Genomes Project was used as the linkage disequilibrium reference panel, aligning with the European origin of GWAS samples.

## Colocalization and false discovery rate

We performed the following analyses to locate shared causal SNPs in a genome-wide scale, and mutually verify IVs in previous MR.

First, we applied Bayesian colocalization (COLOC) analyses to identify SNPs of pleiotropy. Briefly, colocalization analysis examines whether associations detected by MR methods are driven by the same causal variants [31]. And the shared causal variants identified from COLOC is assumed of ‘causality’ exempted from confounding like linkage disequilibrium, where IVs in MR may confer [30]. Given that COLOC itself could not distinguish vertical or horizontal pleiotropy [30], we searched biological pathway databases (KEGG https://www.kegg.jp) to examine whether the SNPs identified were involved in multiple biological pathways [30]. Specifically, if genes were mapped to one certain biological pathway instead of multiple pathways, the SNPs identified by COLOC are suspected to function via vertical pleiotropy and are thus more likely to be valid IVs in MR. The candidate SNPs identified from COLOC were verified with IVs used in previous MR to finally prioritize SNPs of vertical pleiotropy. In essence, the Bayesian approach COLOC assumes that (1) in each test region, there exists at most one causal SNP for either trait; (2) the probability that a SNP is causal is independent of the probability that any other SNP in the genome is causal; and (3) all causal SNPs are genotyped or imputed and included in the analysis. According to these assumptions, there are five mutually exclusive hypotheses for each test region: (1) there is no causal SNP for either trait (H_0_); (2) there is one causal SNP for trait 1 only (H_1_); (3) there is one causal SNP for trait 2 only (H_2_); (4) there are two distinct causal SNPs, one for each trait (H_3_); and (5) there is a causal SNP common to both traits (H_4_) [31]. Our primary interest lied in the last hypothesis, H_4_ colocalization. Support for each of the hypotheses was quantified by the posterior probability (PP), denoted by PP_0_, PP_1_, PP_2_, PP_3_ and PP_4_ accordingly. These PPs were calculated from the priors and the approximate Bayes factors. We set the prior probability of each SNP that is causal to either of the traits to 1 × 10^−4^ (i.e., one in 10,000 SNPs in the genome are causal to either trait) and causal to both traits to 1 × 10^−6^ (i.e., one in 100 SNPs in the genome causal to one trait are causal to both traits). We used the GWAS summary statistics of COVID-19 and CHD to approximate the Bayesian factors. Testing for colocalization was performed with the R package coloc (http://cran.r-project.org/web/packages/coloc).

To further identify SNPs of horizontal pleiotropy that were excluded by MR previously, and also to rigorously provide a genome-wide view of SNPs showing horizontal pleiotropy, we performed cond/conjFDR [32]. We denoted the conjFDR as FDR_trait1&trait2_, which is defined as the posterior probability that a SNP is null for either phenotype or both simultaneously, given that its *P* values for associations with both phenotypes are as small as or smaller than the observed ones. A conservative estimate of conjFDR was given by the maximum between FDR_trait1_|_trait2_ and FDR_trait2_|_trait1_, which required that loci exceeded a conjFDR significance threshold for both phenotypes jointly. SNPs with a conjFDR value less than 0.05 were considered shared loci [32].

### Role of the funding source

The study was supported by the Special Fund for Health Scientific Research in Public Welfare (Grant No. 201502006), the Key Project of Natural Science Funds of China (Grant No. 8123066), the National Natural Science Foundation of China (Grant No. 81872695), the Fujian Provincial Health Technology Project (Grant No. 2020CXB009), the Natural Science Foundation of Fujian Province, China (Grant No. 2021J01352) and the China Postdoctoral Science Foundation (Grant No. BX2021021). These funding sources supported the study as a not-for-profit endeavor.

## Results

### The causal effect of COVID-19 on CHD risks

The bidirectional MR suggested a one-way causal relationship of COVID-19 with CHD (COVID-19 → CHD), with higher COVID-19 susceptibility increasing CHD risks. Each per unit increase in liability to very severe COVID-19 corresponded to an increased risk of CHD (MR_IVW_: OR = 1.01, 95% CI 1.01–1.02,* P* = 2.2E−14). Sensitivity analyses generated consistent effect estimates (Table 1). MR Egger intercept tests did not detect any horizontal pleiotropy outliers. Generally, the four MR methods yielded rather consistent results. However, we found no evidence of reversed causal effects from CHD to COVID-19 (Additional file 1: Table S1).

**Table 1:** Estimated causal effect of very severe COVID-19 on CHD susceptibility

MR method	Interpretation	OR	95% CI	*P* value
Inverse variance weighted	Primary result^a^	1.01	1.01–1.02	2.20E−14
MR Egger	Regression estimate^a^	1.03	1.02–1.04	1.68E−07
Intercept	Intercept test for pleiotropy^b^	0.01	− 0.057 to 0.063	9.42E−01
Weighted median	Consistency^a^	1.00	0.99–1.01	5.56E−02
Simple mode	Consistency^a^	1.04	1.03–1.05	1.48E−06

*MR* Mendelian randomization, *OR* odds ratio, *CI* confidence interval

^a^Unit: the estimated odds ratio for CHD per 1‐unit log odds increase in liability to very severe COVID-19

^b^Unit: average pleiotropic effect of a COVID-19 genetic variant on the odds of CHD

### The genetic correlation between COVID-19 and CHD

As listed in Table 2, the correlation attributable to shared genetic variants accounted for 0.18 (95% CI 0.18–0.19) to 0.23 (95% CI 0.23–0.24). Generally, phenotypic correction of more severe COVID-19 and CHD can be explained by shared genetic variants to a larger extent.

**Table 2:** The estimated genetic correlation between Covid-19 and CHD

	Genetic correlation	95% CI	*P* value
^a^Covid 19_A and CHD	0.23	0.23–0.24	4.65E−28
^b^Covid 19_B and CHD	0.21	0.20–0.22	6.21E−28
^c^Covid 19_C and CHD	0.18	0.18–0.19	1.35E−27

^a^COVID-19_A is defined as very severe respiratory confirmed COVID-19 versus the general population

^b^COVID-19_B is defined as hospitalization for COVID-19 versus the general population

^c^COVID-19_C is defined as a positive COVID-19 diagnosis versus the general population

### Bayesian colocalization analysis

We first included 2,508,363 SNPs that were associated with either COVID-19 or CHD We mapped all the overlapped SNPs between COVID19/CHD to the genomic regions with SNP to Gene (S2G) [33], which could be available at https://alkesgroup.broadinstitute.org/cS2G/. With Bonferroni correction for the multiple testing in inflation of Type I error, the significance threshold was set as 0.05/890 = 5.0E−05. Each of the SNPs and their neighboring SNPs (distance within 200 kb) were then utilized to define a test region. After merging overlapping regions, we tested for colocalization in their respective unique regions. Among these unique test regions, one region on chromosome 3 near *LZTFL1/LOC107986083* displayed suggestive causality shared by both COVID-19 and CHD, with a posterior PP4 exceeding 0.70 (Table 3). Within this region, the SNP Chr3:45859561 (rs10490770, nearest gene *LZTFL1*) exhibited the highest maximum PP4 and was considered the putative causal SNP. Additionally, rs17713054, also located in this region, demonstrated suggestive causality concerning the genetic correlation between COVID-19 and CHD. Notably, rs10490770 was previously identified as an IV in MR analyses. Furthermore, as indicated in the KEGG pathway analysis, *LZTFL1* was not found to be involved in multiple pathways. Considering the LD relationship between rs10490770 with rs10490770, rs17713054 was not selected as an IV. Assuming that at most one causal SNP exists in the gene/region, the presence of two SNPs with high LD and high PP4 suggests that these two SNPs either both play a causal role in the outcome or are in strong LD with a single causal variant. Interestingly, both rs10490770 and rs17713054 exhibited associations with COVID-19 (*P* < 5 × 10^−8^) but not with CHD in their original GWAS (*P* > 0.05). Consequently, the SNPs identified in this study displayed vertical pleiotropy, impacting CHD solely through their influence on COVID-19 risk.

**Table 3:** Bayesian colocalization suggested the shared causal SNPs between COVID-19 and CHD susceptibility

SNP	Closest gene	CHRPOS^b^	Ref Allele	Alt Allele	MAF^c^	PP_4_^d^	P_Covid-19	B_Covid-19	P_CHD	B_CHD
^a^Covid 19_A										
rs10490770	*LZTFL1*	3:45864732	T	C	0.10	0.71	8.96E−45	0.62	0.21	0.71
rs17713054	*LZTFL1*	3:45859651	G	A	0.11	0.69	1.47E−44	0.62	0.36	0.82

^a^COVID-19_A is defined as very severe respiratory confirmed COVID-19 versus the general population

^b^CHRPOS is mapped in Hg19 (GRCh37)

^c^MAF, minor allele frequency, is estimated among individuals of European ancestry within CHD datasets

^d^PP_4_ is the posterior probability of a single causal SNP common to COVID-19 and CHD in the test region

### The cond/conjFDR analysis

**Fig. 2:**
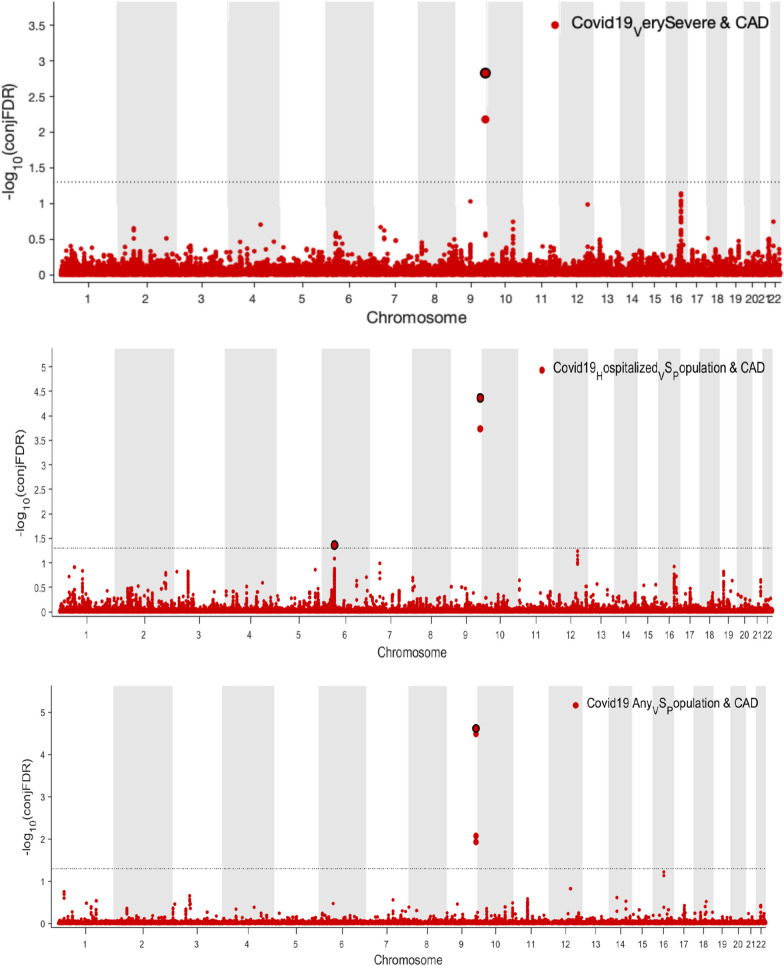
Conjunctional false discovery rate (conjFDR) Manhattan plots of conjunctional − log_10_(FDR) values

To mutually verify the IV validity in MR, and to compensate for the inefficiency in identifying SNPs of horizontal pleiotropy in MR, we further provided a comprehensive, unselected map of shared loci between COVID-19 and CHD with a cond/conjFDR analysis (Fig. 2). With a predefined threshold at conjFDR < 0.05, we identified 5 distinct genetic loci shared between CHD and COVID-19 (Table 4). We observed that 2 SNPs mapped in the *ABO* gene (rs579459 Chr9:136154168 and rs495828 Chr9:136154867) showed consistent signals across varied COVID-19 severities, which implied the value of the *ABO* gene for jointly influencing CHD and COVID-19 (*P* < 5 × 10^–4^).

**Table 4:** The conjunction false discovery rate (conjFDR) suggested evidence for the shared causal SNPs between COVID-19 and CHD susceptibility

SNP	Closest gene	CHRPOS^d^	Ref Allele	Alt Allele	Z Score		Conjunctional false discovery rate	*P* value	
					Covid 19	CHD		Covid 19	CHD
^a^Covid 19_A									
rs579459	ABO	9: 136154168	C	T	4.14	5.17	6.61E−03	3.47E−05	2.30E−07
rs495828	ABO	9: 136154867	T	G	4.48	5.11	1.48E−03	7.52E−06	3.18E−07
^b^Covid 19_B									
rs579459	ABO	9: 136154168	C	T	4.88	5.17	1.84E−04	1.04E−06	2.31E−07
rs495828	ABO	9: 136154867	T	G	5.16	5.11	4.31E−05	2.41E−07	3.19E−07
rs2744961	ILRUN	6:34655000	T	C	3.64	3.64	4.34E−02	2.67E−04	2.67E−04
^c^Covid 19_C									
rs579459	ABO	9: 136154168	C	T	8.13	5.18	2.43E−05	4.31E−16	2.24E−07
rs495828	ABO	9: 136154867	T	G	7.89	5.12	3.27E−05	2.94E−15	3.10E−07
rs4962153	CACFD1	9:136323754	A	G	4.00	3.83	1.17E−02	6.22E−05	1.30E−04
rs3094379	CACFD1	9:136334910	T	C	3.95	4.11	8.43E−03	7.69E−05	3.97E−05

^a^COVID-19_A is defined as very severe respiratory confirmed COVID-19 versus the general population

^b^COVID-19_B is defined as hospitalization for COVID-19 versus the general population

^c^COVID-19_C is defined as a positive COVID-19 diagnosis versus the general population

^d^CHRPOS is mapped in Hg19 (GRCh37)

To observe increments of SNP enrichment for CHD as a function of the significance of COVID-19, a conditional QQ plot was presented (Additional file 1: Fig. S2). Gradual leftward curves indicated that the proportion of nonnull SNPs varies considerably across different levels of association with CHD, which supports the polygenic overlap between these phenotypes.

## Discussion

In the current study, we observed a single-way causal effect of COVID-19 exerted on CHD. Shared genetic variants contributed to the causality, where rs10490770 in *LZTFL1* suggested direct causality (SNPs → COVID-19 → CHD), and SNPs in *ABO* (rs579459, rs495828), *ILRUN* (rs2744961)*,* and *CACFD1*(rs4962153, rs3094379) may simultaneously influence their risks.

To date, limited evidence is available in terms of the causality between COVID-19 and CHD. The current study indicated that the genetically determined risk of COVID-19 infection contributed to higher CHD complication risk. Several observational studies reported adverse CHD outcomes after COVID-19 infection [12, 34], which was consistent with our findings. A recent review also reported a considerable proportion of patients who recovered from COVID-19 continued to experience complications including CHD, even in the absence of a detectable viral infection [8]. This condition, which is often referred to as ‘post-acute COVID-19’ or ‘long COVID’, has been the major concern of clinical care for COVID-19 patients [35]. However, results of most observational studies might be confounded and/or influenced by reverse causality [36]. We thus attempted to infer causality with an alternative method, i.e., bidirectional MR [25–27]. For the reliability of causality observed in our study, we made multiple attempts to provide a rather robust estimate that was less likely to be false positive, for example, multiple sensitivity analyses, and IV validity check in an integrated framework. To be specific, the SNP (rs10490770) identified in colocalization analysis was suspected of vertical pleiotropy with its biological role validated from KEGG. It is suggested that *LZTFL1*, where rs10490770 was mapped, was involved in merely one pathway known to date, namely ‘BBSome-mediated cargo-targeting to cilium’ pathway. No additional evidence was shown regarding its direct association with CHD. Meanwhile, we noted that rs10490770 functioned exactly as IV, which strengthened our beliefs regarding its direct causality of SNP → COVID-19 → CHD. Regarding the relatively moderate effect size, the causality estimate was assumed to be attenuated compared to the true causal effect*,* for the limited number of genetic variants that overlapped between the COVID-19 HG meta-analysis and the datasets that were used for this study. Results should be interpreted with caution in terms of the genetically determined causality between the CHD incidence and COVID-19. In essence, the merit of MR limited its estimation of causality to the genetically determined risk of COVID-19 and CHD risks. Given that the genetic susceptibility of COVID-19 accounted for only a certain proportion of its total phenotypic variation, true causal estimates between these two traits were supposed to be much larger.

As the causality inferred previously may involve an interplay of both genetic and environmental factors, one cannot decide whether the causality inferred previously is contributed from their shared genetic variants. In this context, quantifying the genetic correlation can be thought as a prerequisite for subsequent identification of shared causal SNPs. In the current study, the genetic correlation was estimated at 0.18(COVID-19_C) to 0.23(COVID-19_A), suggesting an increasing trend between CHD and more severe COVID-19. However, it should be noted that the strength of their genetic correlation might be overestimated, as this analysis covers the entire genome. Still, it motivated us to further locate where these shared causal SNPs lied precisely in the genome. Generally, the genetic correlation is thought to be attributable to either vertical pleiotropy or horizontal pleiotropy [37]. Therefore, we applied COLOC coupled with cond/conj FDR to provide a comprehensive view of shared causal SNPs in a genome-wide scale. By integrating corresponding epidemiologic findings from populations with understanding of their biological functions, these SNPs identified may advance current knowledge of underlying mechanisms and may also facilitate clinical care of ‘Long COVID-19’ [35].

Commonly, COVID-19 and CHD are hypothesized to be linked by several biological mechanisms, including immune response, and endothelial dysfunction [21, 38, 39]. Of note, most SNPs identified in the current study were mapped in these pathways. Two SNPs (rs579459 and rs495828) in the *ABO* gene were assumed to be promising putative causal loci, as they showed consistent signals of pleiotropy when assessing CHD and COVID-19 of the three severity types. Recent studies showed that the *ABO* gene, located in 9q34.2, which determines blood type, may affect COVID-19 disease severity [40]. Several observational studies further reported a relationship between ABO blood groups and adverse CHD complications post COVID-19 [19, 20, 38]. In a recent GWAS meta-analysis, where investigators sampled 1980 patients with COVID-19-related respiratory failure and analyzed 8582968 SNPs, further cross-replicated the association of rs657152 at locus 9q34 with the COVID-19 severity [41]. In the current study, it is noticed that rs657152 was in linkage disequilibrium with rs579459 (*D*′ = 0.99), and they both were found to be expression qualitative trait loci (eQTLs) responsible for immune stimulation upon regulatory variant activity (http://pubs.broadinstitute.org/mammals/haploreg/haploreg.php). The activation of the immune response was assumed to be the key for both CHD risks and viral clearance [42]. On the one hand, both rs579459 and rs495828 were repetitively underscored to be associated with CHD risks, where immune response was suspected in the pathology [43–45]. On the other hand, both rs579459 and rs495828 functioned as eQTL [46] responsible for immune response activation and thus were hypothesized to influence the COVID-19 outcomes [47]. An alternative hypothesis is Renin-Angiotensin-System (RAS) unbalancing, where RAS-pathway genes, including rs495828 in *ABO,* was suspected predictive of CHD complications of COVID-19 [48]. Moreover, both rs579459 and rs495828 were found to be associated with Motifs change in Nkx2, where autoimmune mechanism underlying the acute respiratory distress syndrome in SARS-COV-2 was undergirded [47]. Considering Motifs play important regulatory roles, and may bind SARS-CoV-2 spike protein [49], rs579459 and rs495828 identified in the current study are worthy of further replication to fully clarify their roles.

For rs10490770 located near *LZTFL1* in the 3p21.31 region, it was in high LD with rs17713054 (*D*′ = 1), which was identified in COVID-19 GWASs as conferring a twofold increased risk of respiratory failure [5, 41, 50, 51] and an over twofold increased risk of mortality for individuals under 60 years old [52]. Also, rs10490770 was in LD with rs11385942 (*D*′ = 0.98) which was cross-validated in a recent COVID-19 GWAS meta-analysis [41]. However, despite repetitive statistical significance reported in the 3p21.31 region, its specific role in COVID-19 infection remained unexplained [50, 51]. We coincidentally observed rs17713054 in this region colocalized between COVID-19 and CHD, which might offer insights for further uncovering the biological role in this region by simultaneously taking COVID-19 and its CHD complication into considerations. Recent studies reported that rs17713054-affected enhancer upregulates *LZTFL1* [16, 17], which is currently known to be actively involved in the epithelial–mesenchymal transition in the viral response pathway of COVID-19 [50]. We further supplemented eQTL colocalization analysis in the 3p21.31 region (data not shown in the main text). We confirmed that rs10490770 colocalized with eQTL and was highly expressed in the lung tissue (Additional file 1: Figure S2). Meanwhile, no known evidence showed rs10490770 was directly associated with CHD, nor with its belonging pathway ‘BBSome-mediated cargo-targeting to cilium’ pathway. Evidence gathered thus far consistently supported our findings that rs10490770 function only through COVID-19 to CHD. Although statistical signals are not necessarily validated biological evidence, we provided preliminary hints for further studies to uncover the underlying molecular mechanism.

The current study has several clinical implications. First, this study suggested that genetic predisposition to COVID-19 is a causal risk factor for CHD, leading to the hypothesis that reducing the COVID-19 infection risk or alleviating COVID-19 severity among those with specific genotypes might reduce their subsequent CHD adverse outcomes. Initially recognized as a respiratory system disease, COVID-19 has been found to interact with and affect the cardiovascular system leading to myocardial damage and cardiac and endothelial dysfunction [53]. In fact, cardiac damage has been noted even without clinical features of respiratory disease [54], with new-onset cardiac dysfunction common in this subgroup [55, 56]. Specifically, the CHD incidence secondary to COVID-19 infection might be characterized with massive damage in the vascular endothelium and cardiac myocytes due to the systemic inflammatory response in severe COVID-19, which includes the release of high levels of cytokines (known as cytokine release syndrome) [57–59]. These patients with subsequent myocardial involvement could suffer from several potentially life-threatening symptoms [1]. Second, these SNPs identified may be of clinical implications for identifying the target population who are more vulnerable to adverse CHD outcomes post COVID-19 and may also advance treatments of ‘Long COVID-19’ [35].

This study also had limitations worthy of noting. First, the validity of the genetic instruments was not fully understood. It is possible that the genetic instruments may have an indirect effect on the outcome via a currently unknown pathway that does not involve the risk factor for interest. Nevertheless, we addressed this issue by adopting the MR-Egger intercept, although it cannot be ruled out unequivocally. Second, as GWAS summary datasets were extracted from Europeans, the generalizability of the study results was limited to Europeans only. Third, study participants included in the COVID-19 were not screened for CHD at baseline and vice versa. The presence of outcomes in the exposure dataset may bias the causal estimates in MR analyses. However, this is a general limitation of two-sample MR analyses and is inevitable without individual-level data. Third, we acknowledge the potential bias introduced by environmental factors, which could not be completely mitigated, despite our efforts to adjust for age and gender in the GWAS summary statistics and our multivariate Mendelian randomization analysis. However, our results indicate that our findings remain robust in the presence of some established environmental factors. Fourth, the methods we adopted that were built upon GWAS summary statistics, including the LDSC method, along with the Mendelian randomization and Bayesian colocalization, required larger sample sizes than methods that use individual genotypes to achieve equivalent standard error. Of note, our analyses were limited by the number of genetic variants that overlapped between the COVID-19 HG meta-analysis and the datasets that were used for this study. Thus, we could not test some genes that may be of importance. It is also possible that, with larger sample sizes, the genetic association of COVID-19 severity and CHD could become more significant, and confidence intervals would narrow around true estimates.

## Conclusion

In summary, this study indicated the putative causality of COVID-19 genetic susceptibility on incident CHD. It underlined rs10490770 located near *LZTFL1* and SNPs in *ABO* (rs579459, rs495828), *ILRUN* (rs2744961), and *CACFD1* (rs4962153, rs3094379) may simultaneously influence their risks. Further studies are warranted to clarify their underlying mechanism.

### Supplementary Information

**Additional file 1**. Supplementary Tables and Figures.

## Data Availability

Anonymized research data could be obtained the COVID-19 Host Genetic Initiative (COVID-19 HG) (https://www.covid19hg.org/results/r5/) and the CARDIoGRAMplusC4D consortium (http://www.cardiogramplusc4d.org/).
