# Supplementary material for: Identification of genetic loci jointly influencing COVID-19 and coronary heart diseases
Source: Hum Genomics. 2023 Nov 14;17:101. doi: 10.1186/s40246-023-00547-8 (PMC10647050; doi:10.1186/s40246-023-00547-8)
Supplement: Supplementary file 1 — Additional file 1. Supplementary Tables and Figures. [file 40246_2023_547_MOESM1_ESM.docx]

Content

[S-Table 1. Description of the COVID-19 and CHD data profiles 2](#_Toc147704317)

[S-Table 2. Estimated causal effect of CHD on very severe COVID-19 susceptibility 3](#_Toc147704318)

[S-Table 3. A Multivariable Mendelian Randomization study on the Estimated causal effect of CHD on COVID-19 susceptibility 3](#_Toc147704319)

[Supplementary Figure S1. Polygenic Overlap between COVID-19 and CHD 4](#_Toc147704320)

[Supplementary Figure S2. A Bayesian colocalization of 3p21.31 region and eQTL analysis evidencing the role of *LZTFL1* in influencing susceptibility to COVID-19 4](#_Toc147704321)

**Supplementary Table 1**

## S-Table 1. Description of the COVID-19 and CHD data profiles

| **Trait of interest** | **GWAS summary statistics** | **Study Population** | **Case/Controls (N)** | **SNP number (N)** | **Diagnosis** |
| --- | --- | --- | --- | --- | --- |
| **COVID-19** | COVID-19 Host Genetic Initiative, Release of Round 5 (https://www.covid19hg.org/results/r5/) | GWAS meta-analyses composing of 49 prospective/retrospective/case-control studies from the UK, America, Netherlands, Estonia, etc. |  |  |  |
| COVID-19_A |  |  | 5,101 /1,383,241 | 9,856,860 | very severe respiratory confirmed COVID-19 versus the general population |
| COVID-19_B |  |  | 9,986 /1,877,672 | 8,152,415 | the hospitalized versus the general population |
| COVID-19_C |  |  | 38,984 /1,644,784 | 8,738,878 | a positive COVID-19 diagnosis versus the general population |
| **CHD** | the CARDIoGRAMplusC4D consortium (http://www.cardiogramplusc4d.org/) | GWAS meta-analyses composing of 22 case-control studies of Europeans | 22,233 /64,762 | 2,419,895 | Case status was defined by an inclusive CHD diagnosis (e.g., myocardial infarction, acute coronary syndrome, chronic stable angina, or coronary stenosis >50%) |

**Supplementary Table 2**

## S-Table 2. Estimated causal effect of CHD on very severe COVID-19 susceptibility

| **MR method** | **Interpretation** | **OR** | **95% CI** | ***P* value** |
| --- | --- | --- | --- | --- |
| Inverse variance weighted | Primary result ^a^ | 1.01 | 0.97-1.06 | 0.595 |
| MR Egger | Regression estimate ^a^ | 1.02 | 0.92-1.13 | 0.757 |
| intercept | Intercept test for pleiotropy ^b^ | 0.00 | -0.04-0.04 | 0.956 |
| Weighted median | Consistency ^a^ | 1.00 | 0.97-1.04 | 0.866 |
| Simple mode | Consistency ^a^ | 1.03 | 0.98-1.00 | 0.302 |

Abbreviations: MR: Mendelian randomization; OR, odds ratio; CI, confidence interval

^a^ Unit: the estimated odds ratio for severe COVID-19 per 1‐unit log odds increase in liability to very severe CHD.

^b^ Unit: average pleiotropic effect of a CHD genetic variant on the odds of very severe COVID-19.

**Supplementary Table 3**

## S-Table 3. A Multivariable Mendelian Randomization study on the Estimated causal effect of COVID-19 susceptibility on CHD

| **MR method** | **Interpretation** | **OR** | **95% CI** | ***P* value** |
| --- | --- | --- | --- | --- |
| Inverse variance weighted | Primary result ^a^ | 1.01 | 1.01-1.03 | 1.82E-10 |
| MR Egger | Regression estimate ^a^ | 1.03 | 1.02-1.04 | 3.69E-06 |
| intercept | Intercept test for pleiotropy ^b^ | 0.00 | -0.04-0.05 | 0.893 |
| Weighted median | Consistency ^a^ | 1.00 | 0.99-1.02 | 6.62E-02 |

Abbreviations: MR: Mendelian randomization; OR, odds ratio; CI, confidence interval. The multivariable mendelian randomization study adjusted for the BMI and type 2 diabetes.

^a^ Unit: the estimated odds ratio for severe COVID-19 per 1‐unit log odds increase in liability to very severe CHD.

^b^ Unit: average pleiotropic effect of a CHD genetic variant on the odds of very severe COVID-19.

**Supplementary Figure S1**
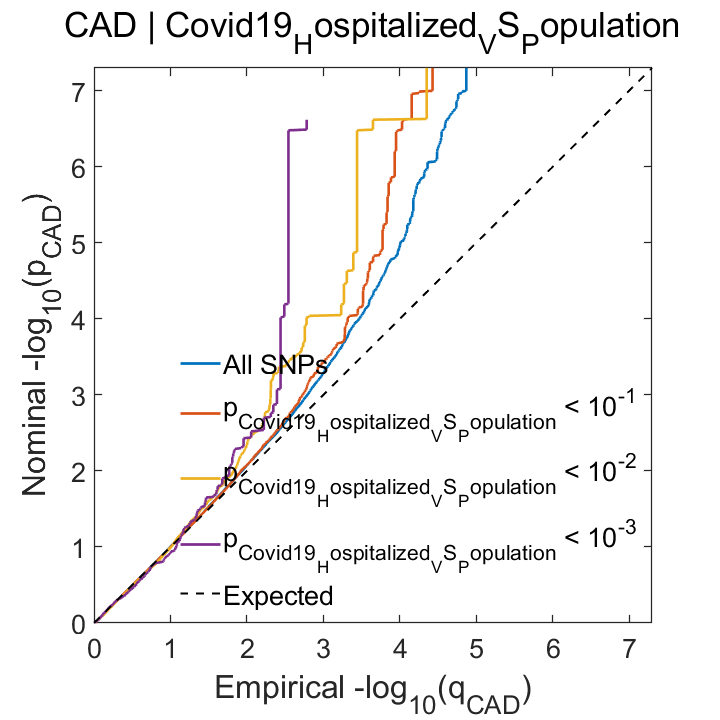

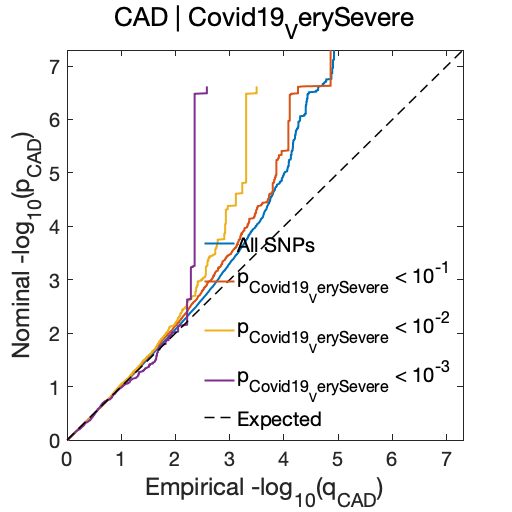

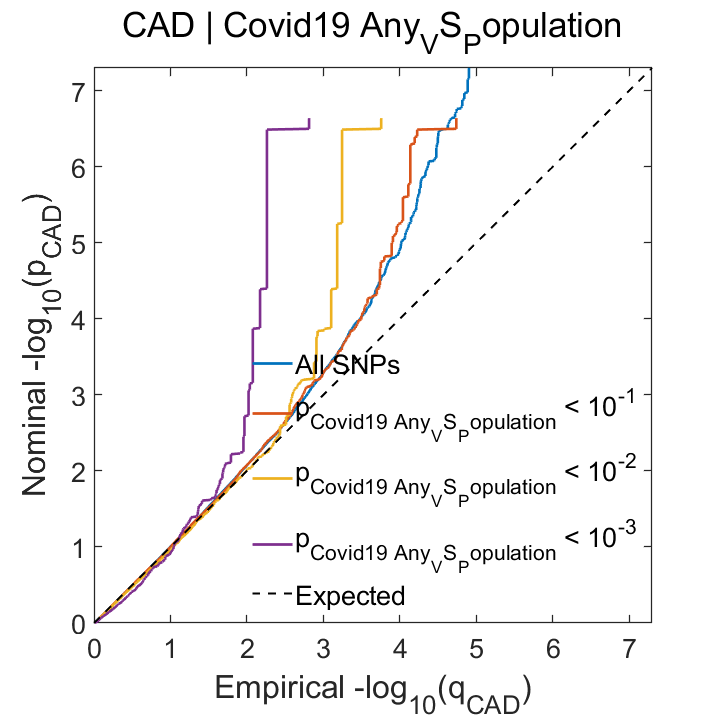


| A B C |
| --- |
| Supplementary Figure S1. Polygenic Overlap between COVID-19 and CHD |

**Supplementary Figure S
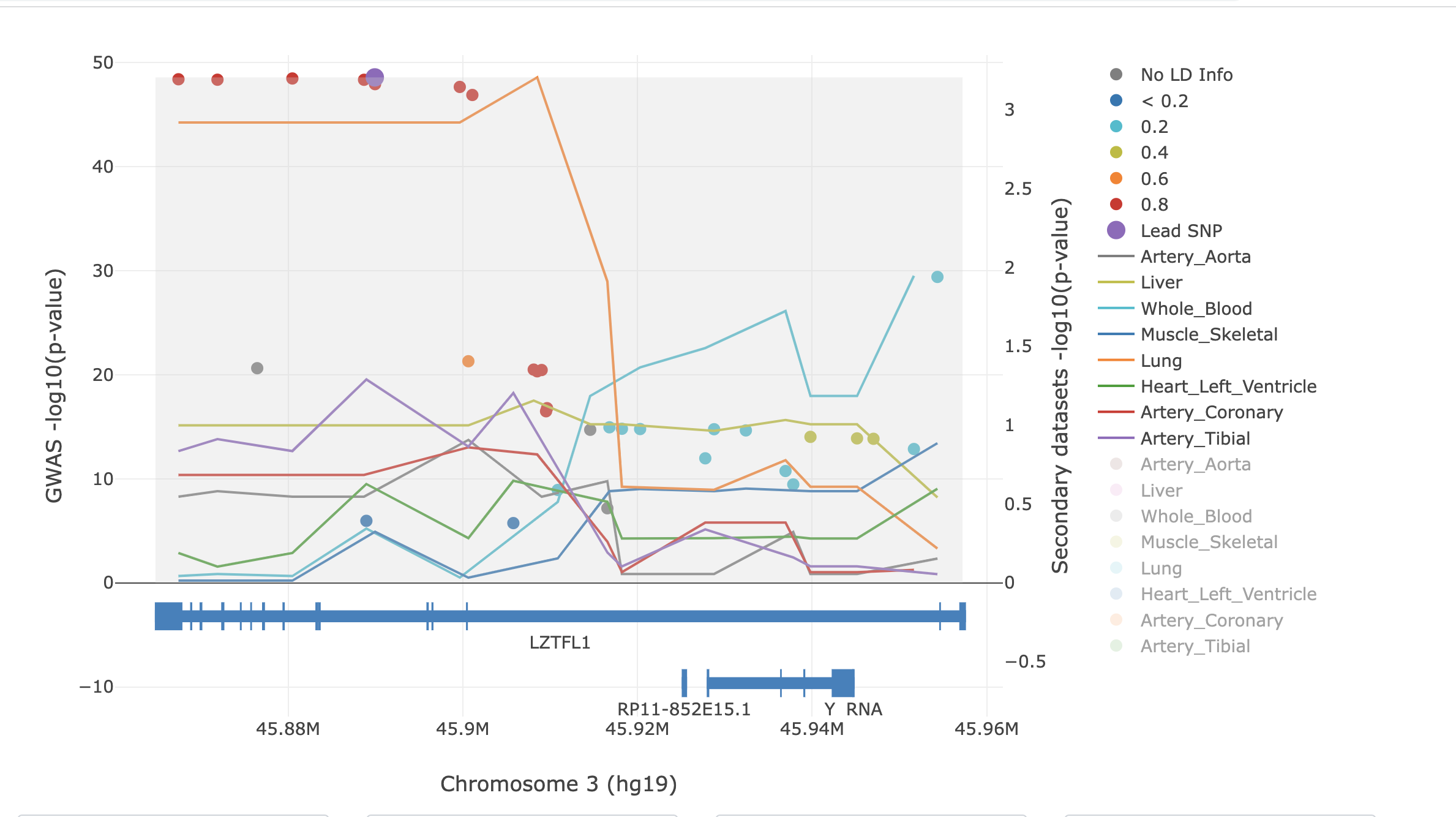
2**

## Supplementary Figure S2. A Bayesian colocalization of 3p21.31 region and eQTL analysis evidencing the role of *LZTFL1* in influencing susceptibility to COVID-19
